# Antioxidants and Obesity

**DOI:** 10.3390/ijms241612832

**Published:** 2023-08-16

**Authors:** Francesca Bonomini

**Affiliations:** 1 Division of Anatomy and Physiopathology, Department of Clinical and Experimental Sciences, University of Brescia, 25123 Brescia, Italy; francesca.bonomini@unibs.it; Tel.: +39-030-371-7477; 2 Interdepartmental University Center of Research “Adaptation and Regeneration of Tissues and Organs (ARTO)”, University of Brescia, 25123 Brescia, Italy

Obesity and its global prevalence are increasingly becoming serious worldwide risks. Extensive evidence to date has suggested that an excess of body fat is linked to an increase in markers of oxidative stress. Indeed, oxidative stress plays a crucial role in the development of obesity-related diseases, such as cardiovascular diseases, diabetes, cognitive dysfunction, cancer, and decreased fertility. Moreover, recent data have demonstrated that increased fat deposition may be related to lower-than-normal levels of antioxidants in the body.

This Special Issue in the *International Journal of Molecular Sciences*, entitled “Antioxidants and Obesity”, includes a total of nine contributions (five original articles and four reviews) providing new information about the pathogenesis and molecular mechanisms underlying obesity and its related diseases, as well as natural therapies and perspectives regarding their treatment. Oxidative stress can be the primary cause or a secondary effect of many human disorders, including obesity-related disorders.

Li et al. [1] reviewed the current state of research on the interplay of inflammation and oxidative stress in obesity and insulin resistance. Moreover, they focused on research on the role of Nuclear factor E2 related factor 2 (Nrf2), in obesity-related inflammation and oxidative stress conditions. In particular, Nrf2 responds by increasing antioxidant transcription levels, restoring tissue and organ homeostasis. Therefore, Nrf2 has emerged as a critical target for counteracting insulin resistance and obesity. The authors also evaluated the Nrf2 pathway in different conditions and considered the potential use of Nrf2 activators for the treatment of insulin resistance.

Lewandowski et al. [2] investigated the alterations in the concentration/activity of superoxide dismutase isozymes (SODs) in human plasma, in the context of sex, obesity, exposure to cigarette smoke, and genotypic variability of five selected single nucleotide polymorphisms in genes SOD1, SOD2, and SOD3. The authors showed that in both men and women, obesity is associated with an increase in values of total antioxidative capacity in serum. In men, there was increased SOD1 concentration and lower SOD3 concentration in plasma compared to women. Moreover, the same sex-specific differences were observed in the concentration of metals, such as copper, zinc, and cadmium. Correlations shown in this study indicate that both total antioxidant capacity and SODs serum levels may adapt to the insulin resistance and inflammation-derived oxidative stress found in obesity.

El-Shehawi et al. [3] studied the relationship between obesity and male fertility by analyzing testicular transcriptomes in an animal model of high-fat-diet-induced obesity. The authors showed that obesity negatively affects male fertility through various routes. It causes the reduction of testosterone due to the increase in cholesterol metabolism, resulting in augmented liver bile acids production in response to the increased fat digestion and to the enhanced testosterone turnover into estradiol in the testes. This ultimately causes a lower testosterone/estradiol ratio that reduces fertility. Moreover, obesity could cause a reduction of sperm chemotaxis via the downregulation of the olfactory receptor genes, which leads to decreased fertility. Fertility is further lowered by obesity through the downregulation of adherens junction components, which in turn cause the premature release of sperm from Sertoli cells.

Jeruzal-Swiatecka et al. [4] reviewed the potential role of taste receptors across a range of clinical conditions. Taste is one of the basic human senses used for the chemical analysis of food composition. Taste recognition begins at the taste receptors, which are found on the mucous of the tongue, palate, and throat but also in the intestines, brain, bladder, lower and upper respiratory tract, and pancreas. The five basic tastes recognized by humans can be linked to ion channels (sour and salty) or transmitted through receptors linked to the G proteins (GPCRs) (sweet, bitter, and umami flavors). The two main types of GPCRs are the Tas1r and TAS2R families. In this review, the authors provided evidence for a significant association between eating behavior, obesity, and diabetes, and TASRs receptors attributed to bitter taste receptors (TAS2Rs), especially TAS2R38.

Emerging research evidence indicates that natural antioxidants can modulate oxidative stress and improve immune function in obesity. Thus, five studies in this Special Issue assessed the role of natural antioxidant compounds in the prevention and treatment of obesity-related diseases.

Bae et al. [5], in their study, identified an anti-obesity peptide from *Allomyrina dichotoma* larvae and investigated the lipid metabolic mechanism. They showed that this peptide affects lipid aggregation in vitro and significantly reduced body weight gain, liver weight, and adipose tissue deposition in an animal model of obesity. Moreover, they showed, in the group treated with the peptide, a decrease in serum levels of total cholesterol, triglyceride, and low-density lipoprotein cholesterol and an increase in high-density lipoprotein cholesterol. Furthermore, this peptide substantially decreased mRNA levels of transcription factors involved in lipid adipogenesis. Taken together, these results suggest that this peptide could be a promising agent for treating and preventing obesity and hypercholesterolemia.

Kato et al. [6], in their research, studied mechanisms of obesity-induced cognitive dysfunction. The authors hypothesized that obesity induces cognitive dysfunction via the acceleration of reactive oxygen species production. Recently, it has been demonstrated that tocotrienols, important members of the vitamin E family, have strong neuroprotective and anti-obesity effects. They studied the potential protective effects of tocotrienol treatment on body weight, brain oxidation levels, and cognitive function in a mouse model of obesity. The results demonstrated that tocotrienols treatment affects body weight gain; moreover, in the brain, it increases the antioxidant capacity and attenuates oxidative stress in the obese mice group. These results suggest a protective effect against obesity and obesity-related cognitive dysfunction for tocotrienols.

Montalbano et al. [7] evaluated the effect of a flavonoid-rich orange (*Citrus sinensis*) juice extract (OJe) in diet-induced obese zebrafish. The results demonstrated that in overfeeding zebrafish, OJe significantly decreased both body weight and body mass index values. Furthermore, it reduced visceral adipose tissue deposition and significantly reduced adipocyte cell size in both visceral and subcutaneous adipose tissues. OJe also demonstrated the ability to modulate some obesity-related genes, such as leptin A, ghrelin, orexin, pro-opiomelanocortin, and neuropeptide Y, in both the gut and brain. Taken together, the results suggest a potential role for the flavonoids found in orange juice in weight management.

Ojulari [8] reviewed a number of studies that reported the effects of natural compounds present in foods, showing advantages over chemical treatments. Seaweed carotenoids have been considered by different researchers due to their remarkable biological activities, especially related to their antioxidant properties. The review collected the research published over the last decade, both in vitro and in vivo studies, on seaweed xanthophyll carotenoids, focusing on fucoxanthin and astaxanthin and their involvement in oxidative stress control and modulation in obesity. The authors also considered the involvement of these carotenoids in the control of major transcription factors and enzymes, and their effects on obesity.

Zielinska-Blizniewska et al. [9] reviewed the role of reactive oxygen species in obesity and the potential use of phytotherapy for treating obesity-related health problems. The authors reported on the anti-obesity effects of plant extracts which were screened in vitro and in vivo using animal and human subjects. The literature examined showed how bioactive compounds derived from plants may be a good natural alternative for weight gain control and a source of numerous biological secondary metabolites, including polyphenols. The review provides evidence, above all, of the potential use of polyphenols as a natural adjunctive therapy and as a good nutritional strategy for treating obesity and its related diseases without noticeable side effects.

## References

[1] Li S., Eguchi N., Lau H., Ichii H. (2020). The Role of the Nrf2 Signaling in Obesity and Insulin Resistance. Int. J. Mol. Sci..

[2] Lewandowski Ł., Kepinska M., Milnerowicz H. (2020). Alterations in Concentration/Activity of Superoxide Dismutases in Context of Obesity and Selected Single Nucleotide Polymorphisms in Genes: *SOD1*, *SOD2*, *SOD3*. Int. J. Mol. Sci..

[3] El-Shehawi A.M., El-Shazly S., Ahmed M., Alkafafy M., Sayed S., Farouk S., Alotaibi S.S., Elseehy M.M. (2020). Transcriptome Analysis of Testis from HFD-Induced Obese Rats (*Rattus norvigicus*) Indicated Predisposition for Male Infertility. Int. J. Mol. Sci..

[4] Jeruzal-Świątecka J., Fendler W., Pietruszewska W. (2020). Clinical Role of Extraoral Bitter Taste Receptors. Int. J. Mol. Sci..

[5] Bae S.M., Fan M., Choi Y.-J., Tang Y., Jeong G., Myung K., Kim B.-g., Kim E.-K. (2020). Exploring the Role of a Novel Peptide from *Allomyrina dichotoma* Larvae in Ameliorating Lipid Metabolism in Obesity. Int. J. Mol. Sci..

[6] Kato Y., Aoki Y., Fukui K. (2020). Tocotrienols Influence Body Weight Gain and Brain Protein Expression in Long-Term High-Fat Diet-Treated Mice. Int. J. Mol. Sci..

[7] Montalbano G., Mania M., Guerrera M.C., Laurà R., Abbate F., Levanti M., Maugeri A., Germanà A., Navarra M. (2019). Effects of a Flavonoid-Rich Extract from *Citrus sinensis* Juice on a Diet-Induced Obese Zebrafish. Int. J. Mol. Sci..

[8] Ojulari O.V., Lee S.G., Nam J.-O. (2020). Therapeutic Effect of Seaweed Derived Xanthophyl Carotenoid on Obesity Management; Overview of the Last Decade. Int. J. Mol. Sci..

[9] Zielinska-Blizniewska H., Sitarek P., Merecz-Sadowska A., Malinowska K., Zajdel K., Jablonska M., Sliwinski T., Zajdel R. (2019). Plant Extracts and Reactive Oxygen Species as Two Counteracting Agents with Anti- and Pro-Obesity Properties. Int. J. Mol. Sci..

